# Cerebrospinal fluid cholinergic biomarkers are associated with postoperative delirium in elderly patients undergoing Total hip/knee replacement: a prospective cohort study

**DOI:** 10.1186/s12871-020-01166-9

**Published:** 2020-09-28

**Authors:** Xu Lin, Jiaming Tang, Chen Liu, Xiaoxuan Li, Xipeng Cao, Bin Wang, Rui Dong, Wei Xu, Xinjuan Yu, Mingshan Wang, Yanlin Bi

**Affiliations:** 1 Department of Anesthesiology, Qingdao Municipal Hospital, 5, Dong-Hai Middle Road, Shi-Nan District, Qingdao, 266071 Shandong Province China; 2 Department of Anesthesiology, Affiliated Hospital of Qingdao University, 59, Hai-Er Road, Lao-Shan District, Qingdao, 266000 Shandong Province China; 3 Department of Anesthesiology, Weifang Medical University, 7166, Bao-Tong West Street, Wei-Cheng District, Weifang, 261053 Shandong Province China; 4 Clinical Research Center, Qingdao Municipal Hospital, 5, Dong-Hai Middle Road, Shi-Nan District, Qingdao, 266071 Shandong Province China; 5 Department of Neurology, Qingdao Municipal Hospital, 5, Dong-Hai Middle Road, Shi-Nan District, Qingdao, 266071 Shandong Province China; 6 Central Laboratory, Qingdao Municipal Hospital, 5, Dong-Hai Middle Road, Shi-Nan District, Qingdao, 266071 Shandong Province China

**Keywords:** Postoperative delirium, Cholinergic, Cerebrospinal fluid, Elderly

## Abstract

**Background:**

Postoperative delirium (POD) is a frequent complication after surgery and its occurrence is associated with poor outcomes. The neuropathology of this complication is unclear, but it is important to evaluate relevant biomarkers for postoperative status. The purpose of this study is to explore the relationship between expression levels of cholinergic biomarkers in cerebrospinal fluid (CSF) and the occurrence and development of POD in elderly patients.

**Methods:**

Four hundred and ninety-two elderly patients aged 65 years old or older with elective total hip/knee replacement received combined spinal-epidural anesthesia. Preoperative baseline cognitive function was assessed using the Mini-Mental State Examination (MMSE) before surgery. Each patient was interviewed in post-anesthesia care unit (PACU) and on the first, second, third and seventh (or before discharge) postoperative days. POD was diagnosed using the Confusion Assessment Method (CAM), and POD severity was measured using the Memorial Delirium Assessment Scale (MDAS). Preoperative CSF and plasma choline acetyltransferase (ChAT), acetylcholinesterase (AChE), butyrylcholinesterase (BuChE), interleukin-6 (IL-6) and tumor necrosis factor-α (TNF-α) levels were determined by ELISA. The levels of ChAT, AChE and BuChE activities were determined by spectrophotometry.

**Results:**

POD was detected in 11.4% (51/447) of the patients. AChE, BuChE, ChAT, TNF-α and IL-6 concentrations in CSF and plasma have higher consistency. In preoperative CSF and preoperative and postoperative plasma, down-regulation of the concentration and activity of AChE and BuChE as well as up-regulation of the concentration and activity of ChAT and the concentrations of IL-6 and TNF-α were observed in patients who developed POD, and the decrease in BuChE was the most obvious. Logistic analysis showed the activities of ChAT, AChE and BuChE in CSF were still related to POD after adjusting for related factors such as sex, age, years of education, height, weight, body mass index (BMI), and American Society of Anesthesiologists (ASA) class. Receiver Operating Characteristic (ROC) curve analysis was conducted to determine the Area Under Curve (AUC) of AChE, BuChE and ChAT activity in CSF was 0.679 (*P* < 0.01), 0.940 (*P* < 0.01) and 0.819 (*P* < 0.01) respectively and found that BuChE activity had the most accurate diagnostic value.

**Conclusion:**

The changes in preoperative activity of AChE, BuChE and ChAT in CSF were associated with the development of POD in elderly patients, and BuChE activity had the greatest diagnostic value, which may be related to central cholinergic degradation. These cholinergic biomarkers might participate in the neuropathology of POD, pending further investigations.

**Trial registration:**

This study was registered at Chictr.org.cn (NO. ChiCTR1900023729) June 9th, 2019. (Retrospectively registered).

## Background

Postoperative delirium (POD) is acute central nervous system dysfunction after surgery in elderly patients, and its common symptoms include decreased clarity of consciousness, visual hallucinations, disorientation, memory disorders, and sleep disorders [1]. POD can develop into long-term or even permanent cognitive dysfunction or dementia, which seriously affects the quality of life, increases the incidence and mortality of long-term complications, and brings heavy family and social burden [2]. POD had a clinical incidence ranging from 4 to 70%, specifically 17% after hip/knee surgery [3]. With the development of social economy and the prolongation of human life, more and more elderly patients receive total hip/knee replacement. However, at the current time, effective prevention and treatment are hampered by lack of knowledge about the neuropathology of POD as well as lack of biomarker(s) to identify the risk for the development of POD. The cholinergic system is considered to play a key role in the development of POD [4].

Cholinergic anti-inflammatory pathway (CAIP) in the cholinergic system is a neural circuit that affects and regulates inflammatory response. Stimulation of the vagus nerve can enhance its release of acetylcholine (ACh), which binds to the α7 nicotinic acetylcholine receptor (α7nAChR), inhibiting inflammatory factors from producing anti-inflammatory response [5]. As we all know, ACh is a classical neurotransmitter in the central and peripheral nervous system. ChAT, a synthetase of ACh, and ChE, a degradation enzyme of ACh (including AChE and BuChE) jointly maintain ACh levels and participate in learning and cognition [6]. It was found that central ACh is a neurotransmitter that interacts with the α7 subunit of α7nAChR expression, which is involved in learning and cognition and can inhibit the occurrence of Alzheimer’s disease (AD) [7].

AChE and BuChE activity is an independent risk factor for POD [4]. Plasma ChE activity may be a candidate biomarker for POD in the elderly Chinese population [8]. A meta-analysis showed, CSF biomarkers in POD patients were of more clinical significance and diagnostic value than those in plasma [9]. However, whether CSF cholinergic biomarkers are associated with POD remains unknown.

So the purpose of this prospective cohort study is to evaluate whether the changes in preoperative activity of CSF cholinergic biomarkers were associated with POD. We hypothesized that changes in preoperative activity of these CSF cholinergic biomarkers would be associated with the development of POD. The findings of this investigation may be helpful to prove a correlation between changes in preoperative activity of CSF cholinergic biomarkers and development of POD, which would facilitate further to investigations into the role of CSF cholinergic biomarkers in the neuropathology of POD.

## Methods

### Study design

This was a prospective cohort study to assess whether the changes in preoperative activity of CSF cholinergic biomarkers were associated with POD. The study was approved by the Research Ethics Committee of Qingdao Municipal Hospital and registered at Chictr.org.cn (NO. ChiCTR1900023729).

### Participants

We selected eligible patients who were at least 65 years old and scheduled to have total hip/knee replacement under spinal-epidural joint anesthesia, between December 2018 and December 2019, in the Qingdao Municipal Hospital.

Exclusion criteria (1) Mini-Mental State Examination (MMSE) scores of 23 or less; (2) medical history serious mental or nervous system diseases, such as: Alzheimer’s disease, Parkinson’s syndrome, cerebrovascular accident and cerebrovascular disease;; (3) drug abuse or abuse of psychotropic durgs, long-term use of steroids, and hormone drugs; (4) ASA score [a global score that assesses the physical status of patients before surgery, ranging from 1 (normal health) to 5 (moribund)] [9] greater than 3; (5) preoperative combined with stage III-IV hepatic encephalopathy; (6) severe visual and hearing disorders; (7) unwillingness to comply with the protocol or procedures.

**Fig. 1:**
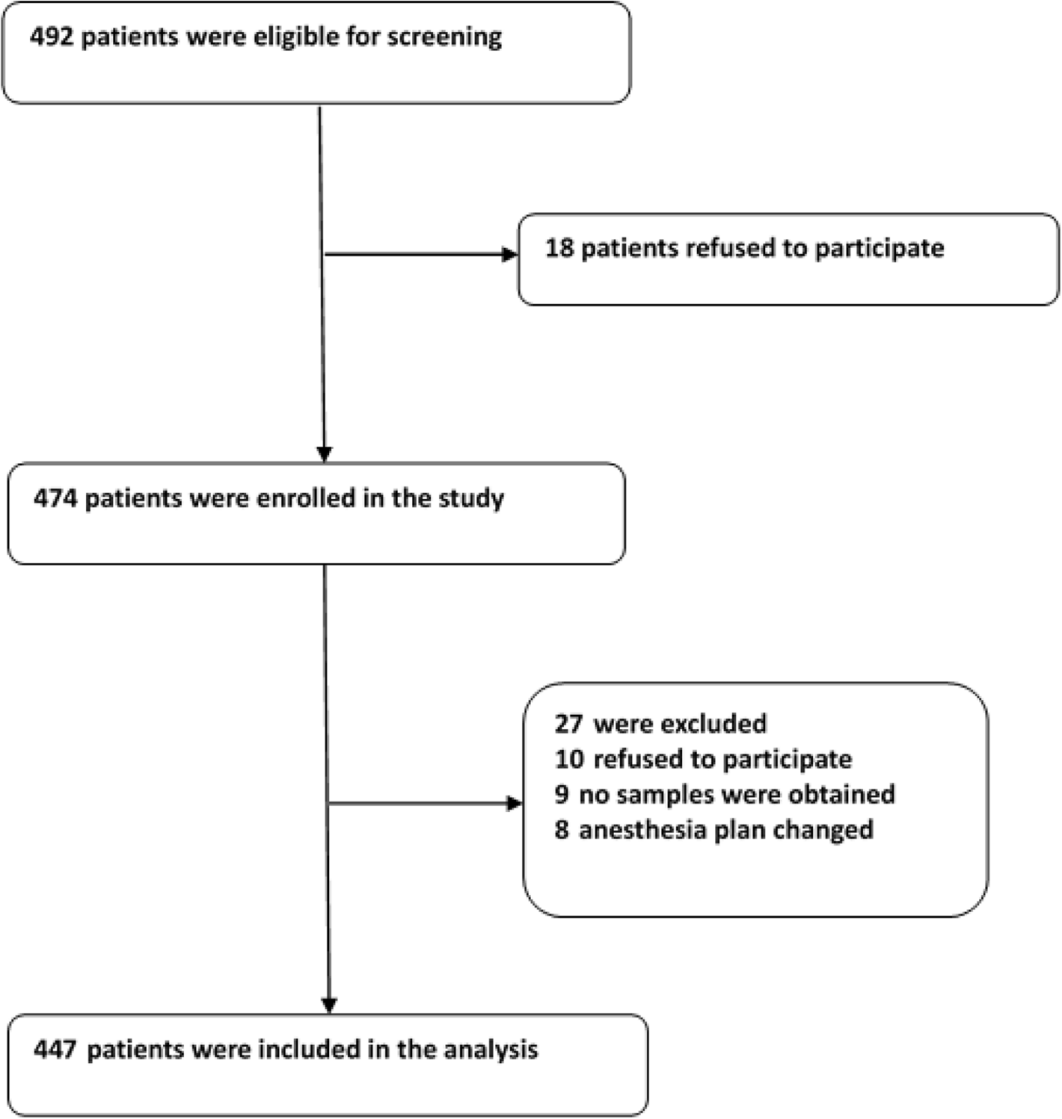
Flow diagram. The flow diagram shows that 492 patients were initially screened for the studies, and 447 patients were finally included in the data analysis

Data of 492 patients were analyzed in this study (see Fig. 1, flow diagram).

### Neuropsychological testing

Participants received interview preoperatively and in PACU, on the first, second, third and seventh (or before discharge) postoperative days. The MMSE was used on the day before the scheduled surgery by neurologist The assessment of delirium was performed in PACU, on the first, second, third and seventh days (or before discharge) after surgery between 9:00 am and 11:00 am by anesthesiologist. We used the visual analog scale (VAS) score of 0–10 (lower score indicating lower level of pain [10]) to assess pain at the same time. POD was defined by the Confusion Assessment Method (CAM) [11, 12], and POD severity was measured using the Memorial Delirium Assessment Scale (MDAS) [11, 12]. The CAM and MDAS in Chinese research has been proven to have good reliability and validity in the Chinese elderly population [13, 14]. Therefore, CAM and MDAS scores positive patients postoperatively in PACU and on the first, second, third and seventh days (or before discharge) were recorded.

### Anesthesia and surgery

Each patient who underwent total hip or total knee replacement under combined spinal and epidural anesthesia uses the same surgical team to avoid the impact of different surgical techniques. During anesthesia, oxygen saturation, electrocardiography, pulse oximetry and non-invasive blood pressure were continuously monitored and were recorded at fixed intervals of 3 min. Three hundred and sixty patients received dexmedetomidine during the surgery for sedation. We used and recorded patient controlled intravenous analgesia for postoperative analgesia. If the patients need it, they were given non-opioid drugs for analgesia, which was recorded.

### Sample collection

Before anesthesia and 24 h postoperatively, peripheral venous blood samples (5 ml) were drawn into EDTA anticoagulant tubes from the enrolled patients. Then, plasma specimens were isolated by two steps of centrifugation. We reversed the anticoagulant tube back and forth 10–12 times so that the blood and anticoagulant could mix well. And the blood samples were centrifuged immediately at 2000 g for 10 min at room temperature for the collection of plasma [15], which were stored at − 80 °C until further analysis.

The CSF (2 ml) was collected in a polypropylene centrifugal tube during spinal-epidural joint block prior to administration of the local anesthetic. The samples were centrifuged immediately at 2000 g for 10 min at room temperature [16, 17], which were stored at − 80 °C until further analysis.

### Elisa

The concentrations of ChAT, AChE, BuChE, TNF-α and IL-6 were detected from 1.5 ml CSF and 1.5 ml plasma using ChAT (Cloud-Clone Corp, Wuhan, China Lot:L190321380), AChE (Cloud-Clone Corp, Wuhan, China Lot:L190321390), BuChE (Cloud-Clone Corp, Wuhan, China Lot:L190321377),TNF-α (Cloud-Clone Corp, Wuhan, China Lot:L190109552) and IL-6 (Cloud-Clone Corp, Wuhan, China Lot:L190117600) assay kit in accordance with the manufacturer’s protocol. Finally, the optical density value (O.D. value) of each hole was measured at the wavelength of 450 m with an enzyme marker [18] (PerkinElmer, EnSpire, USA).

### Spectrophotometry

The activities of ChAT, AChE and BuChE was detected by spectrophotometry from 1.5 ml CSF and 1.5 ml plasma using ChAT (Jiancheng, Nanjing, China A079–2), AChE (Jiancheng, Nanjing, China A024) and BuChE (Jiancheng, Nanjing, China A025) assay kit in accordance with the manufacturer’s protocol. Finally, the absorbance values of each tube were measured at wavelengths of 324 nm and 412 nm using the spectrophotometer [16–18] (NanoDrop Technologies, Wilmington, USA).

### Sample size estimation

The PASS11.0 software was used to estimate the required sample size [19]. The expected sensitivity was 0.9, the allowable sensitivity error was 0.05, the expected specificity was 0.5, the allowable specificity error was 0.05, α was 0.05, and 1–β = 0.8. The two-sided and missed visit ratio was 0.1. The calculated sample size was 447. According to the exclusion criteria, 447 patients were finally included.

### Statistical analysis

We use the mean ± standard deviation (SD), the median and interquartile range (IQR, 25–75 percentile) or a number (%) to express the data. The POD incidence is expressed as a percentage. Therefore, we test the normality of all the variables by the Kolmogorov–Smirnov method. Two independent samples T test were used for intra-group and inter-group comparison. Chi-square test was used to compare the counting data. ICC analysis was used for consistency analysis between CSF and plasma index. Logistic regression was used to analyze the correlation between the activities of ChAT, AChE and BuChE in CSF and POD, and other POD-related influencing factors (such as sex, age, years of education, height, weight, body mass index (BMI), ASA class) were adjusted to verify whether the three were still related to POD, and the odds ratio (OR) and 95% confidence interval (CI) of each factor were calculated. ROC curve was used to analyze the activities of ChAT, AChE and BuChE in CSF. Statistical significance was set at *P* < 0.05. SPSS statistical software, version 21.0 (SPSS, Inc., Chicago, IL, USA), and GraphPad Prism software, version 6.01 (GraphPad Software, Inc., La Jolla, CA, USA), were used for data analysis.

## Results

### Participant characteristics

We included 492 participants, 474 of which met the requirements of this study, and 27 participants were excluded. The reasons for dropouts are shown in Fig. 1. Therefore, 447 patients (*n* = 447) remained for analysis. The demographic and clinical data of the participants are summarized in Table 1.

**Table 1:** The length of anesthesia was defined from the time that the anesthesiologists started the spinal anesthesia in the patients to the time when the patients were sent to the post-anesthesia care unit. The length of surgery was defined from the time of initial incision to the time of the closure of the skin. POD, postoperative delirium; ASA, American Society of Anesthesiologists; cm, centimeter; min, minute; kg, kilogram; ml, milliliter; SD, standard deviation; CSF, cerebrospinal fluid

Characteristics of participants.		
	POD(*N* = 51)	Non-POD(*N* = 396)
Age (year), mean ± SD	73.3 ± 6.5	72.2 ± 6.0
Male, n (%)	23 (45.1)	187 (47.3)
Years of education, n (%)		
0	4 (7.8)	28 (7.1)
1–9	22 (43.1)	168 (42.4)
10–13	12 (23.5)	81 (20.5)
14–17	9 (17.6)	65 (16.4)
> 17	6 (11.8)	54 (13.6)
Height (cm), mean ± SD	163.2 ± 7.1	161.5 ± 6.3
Body weight (kg), mean ± SD	67.4 ± 8.8	65.3 ± 8.7
BMI (kg/m 2), mean ± SD	26.3 ± 3.4	25.1 ± 2.8
ASA class, n (%)		
I	9 (17.6)	63 (15.9)
II	30 (58.8)	231 (58.3)
III	12 (23.5)	102 (25.8)
Time of anesthesia (min), mean ± SD	145.6 ± 19.2	143.2 ± 18.9
Time of surgery (min), mean ± SD	110.3 ± 20.3	108.7 ± 19.1
Type of surgery		
Total hip arthroplasty/replacement, n (%)	30 (58.8)	222 (56.1)
Total knee arthroplasty/replacement, n (%)	21 (41.2)	174 (43.9)
Estimated blood loss (ml), median and 25–75 percentile	180 (125–230)	170 (115–220)
Postoperative the highest CAM score, median and 25–75 percentile	18 (15–27)	
Postoperative the highest MDAS score, median and 25–75 percentile	7 (5–11)	
Postoperative the highest VAS score, median and 25–75 percentile	3 (1–5)	2 (1–3)

We observe the incidence of POD the postoperative assessments was 11.4% (*n* = 51 of the 447 patients). Twenty-three of the 51 patients who developed POD were male. In the patients who subsequently developed POD, the preoperative MMSE score [26.5(25–27)] was not significantly different from the score of those who did not develop delirium [27.1(26–28), *P* = 0.086]. In this study, we found the highest CAM and MDAS scores from the postoperative on the first and second days. Postoperatively, the highest VAS score did not differ between patients with delirium 2(1–3) and without delirium [3(2–5), *P* = 0.180].

Three hundred and sixty patients received dexmedetomidine for sedation during the operation. The mean dose of dexmedetomidine for patients who developed POD (68.6 ± 35.4 μg) was not significantly different from that for participants who did not develop POD (78.2 ± 36.2 μg, *P* = 0.076].

### Consistency between CSF and plasma

**Fig. 2:**
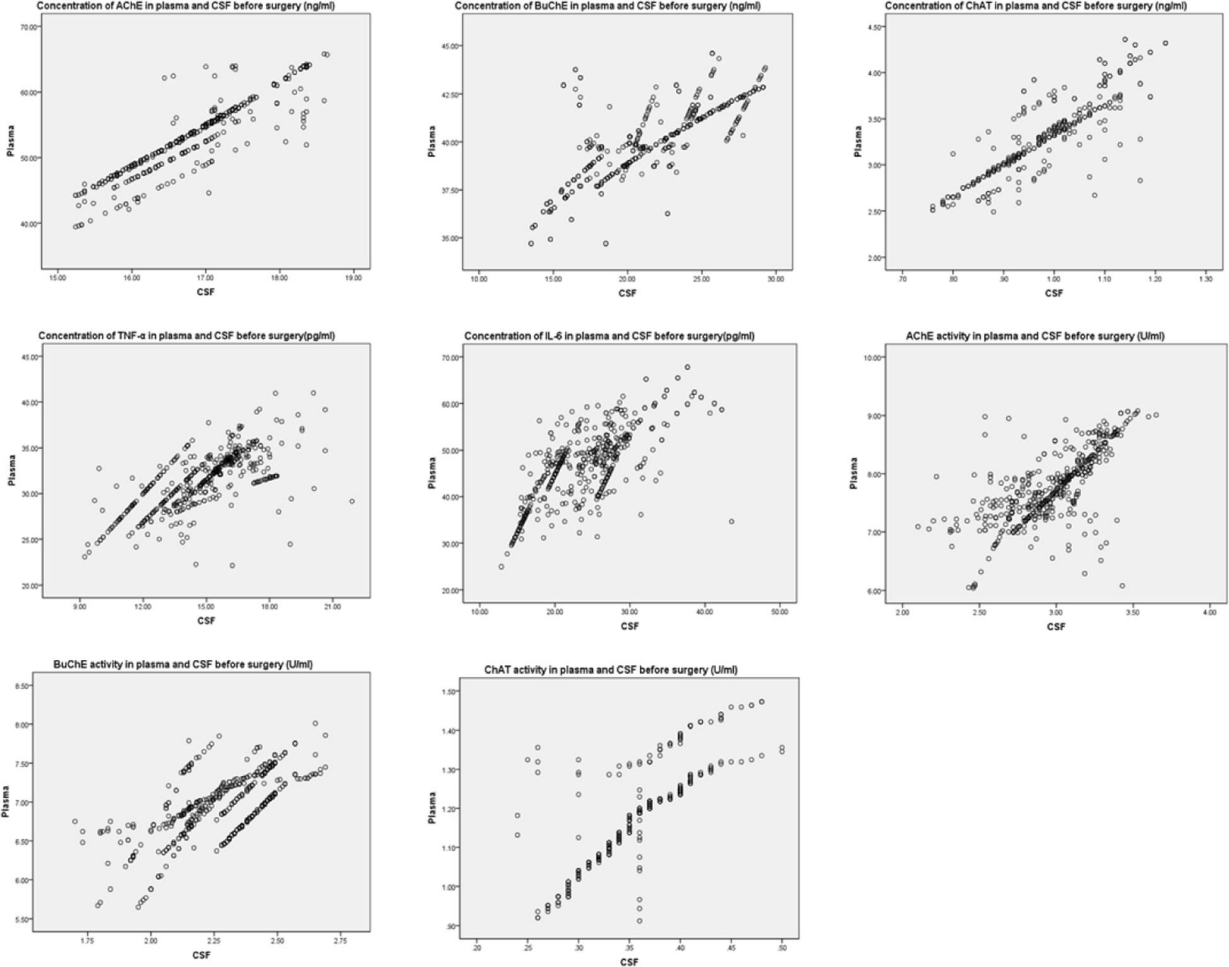
Scatter plots for correlations between preoperative plasma and CSF concentrations of AChE, BuChE, ChAT, TNF-α and IL-6 and scatter plots for correlations between preoperative plasma and CSF activities of AChE, BuChE, ChAT, TNF-α and IL-6 were shown in Fig. 2.(AChE concentration: R = 0.904,BuChE concentration: R = 0.878,ChAT concentration: R = 0.878,TNF-α concentration: R = 0.724,IL-6 concentration: R = 0.702,AChE activity: R = 0.685,BuChE activity: R = 0.659,ChAT activity: R = 0.828)

Spearman correlation analysis showed that the concentrations of AChE, BuChE, ChAT, TNF-α and IL-6 in plasma and CSF had good correlation. Scatter plots for correlations between preoperative plasma and CSF concentrations of AChE, BuChE, ChAT, TNF-α and IL-6 were shown in Fig. 2. Scatter plots for correlations between preoperative plasma and CSF activities of AChE, BuChE, ChAT, TNF-α and IL-6 were also shown in Fig. 2.

### Concentrations of ChAT, AChE, BuChE, IL-6 and TNF-α in preoperative CSF as well as preoperative and postoperative plasma

**Fig. 3:**
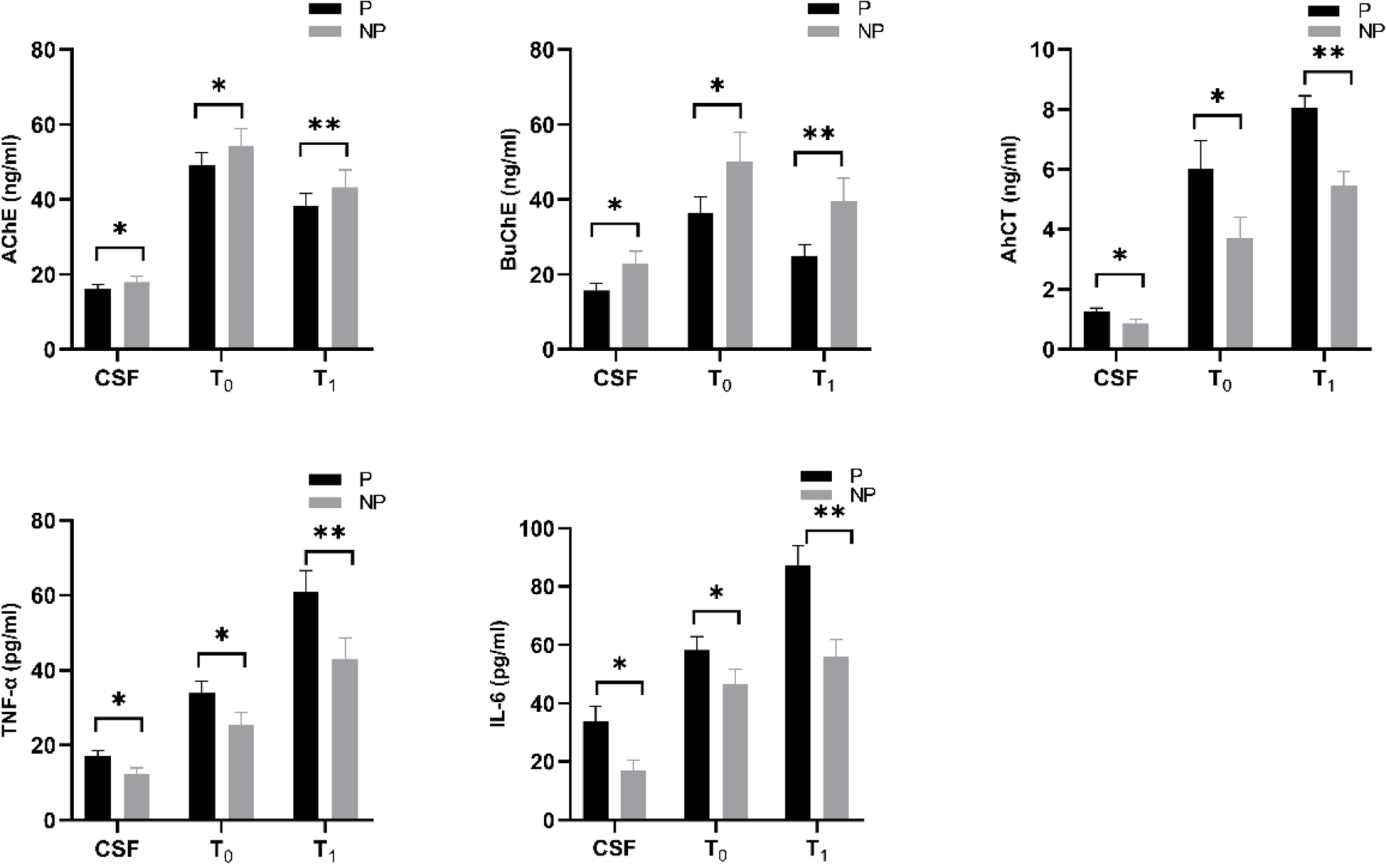
Patients with POD (Group P);Patients without POD (Group NP). Compared with NPOD, ^*a*^*P* < 0.05,Compared with preoperative plasma(T0), ^b^*P* < 0.05, with statistical significance. *P*-values were determined using the t-test. POD, postoperative delirium; CSF, cerebrospinal fluid

Compared with patients without POD, AChE and BuChE concentrations in preoperative CSF as well as preoperative and postoperative plasma in participants with POD were significantly decreased, with BuChE in preoperative CSF having the most significant difference. However, ChAT, IL-6 and TNF-α concentrations were significantly increased. In patients with or without POD, AChE and BuChE in postoperative plasma(T1) were significantly decreased, while ChAT, IL-6 and TNF-α were significantly increased compared with those in preoperative plasma(T0)(Fig. 3).

### Activities of ChAT, AChE and BuChE in preoperative CSF and preoperative as well as postoperative plasma

**Fig. 4:**
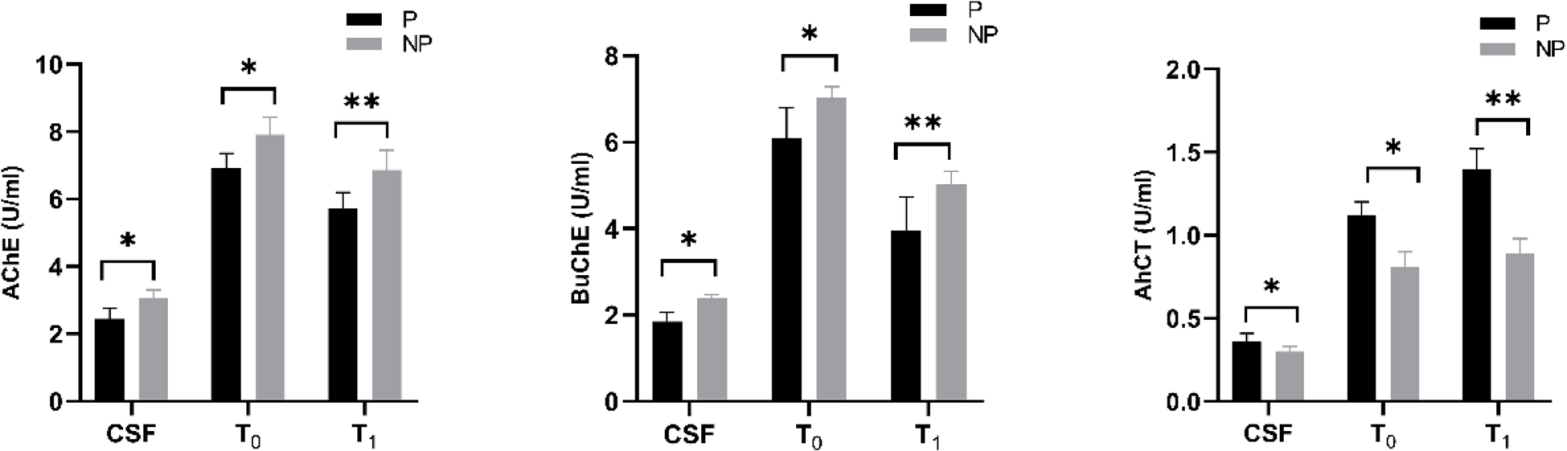
Patients with POD (Group P);Patients without POD (Group NP). Compared with NPOD, ^*a*^*P* < 0.05,Compared with preoperative plasma(T0), ^b^*P* < 0.05, with statistical significance. *P*-values were determined using the t-test. POD, postoperative delirium; CSF, cerebrospinal fluid

Compared with patients without POD, AChE and BuChE activities in preoperative CSF and preoperative and postoperative plasma in the participants with POD were significantly decreased, with BuChE in preoperative CSF having the most significant different. However, ChAT activity was significantly increased. Additionally, in patients with or without POD, AChE and BuChE in postoperative plasma(T1) was significantly decreased, while ChAT was significantly increased, compared with those in preoperative plasma(T0)(Fig. 4).

### ChAT, AChE and BuChE activities in CSF logistic analysis and after proofreading

Logistic analysis of ChAT, AChE and BuChE activities in CSF of all eligible participants showed increased ChAT activity (before adjustion OR 3.828, 95% CI 1.817 ~ 8.330), decreased AChE activity (before adjustion OR 0.016, 95% CI 0.002 ~ 0.063) and decreased BuChE activity (before adjustion OR 0.001, 95% CI 0.000 ~ 0.009) in CSF, after adjustment for POD. After adjustment for other pod-related factors, the logistic analysis showed increased ChAT activity (after adjustion OR 3.886, 95% CI 1.126 ~ 6.605), decreased AChE activity (after adjustion OR 0.009, 95% CI 0.002 ~ 0.059) and decreased BuChE activity (after adjustion OR 0.003, 95% CI 0.000 ~ 0.008), which still correlated with POD (Table 2).

**Table 2:** Adjusted by sex, age, years of education, height, weight, body mass index (BMI), and ASA class

Activity (U/ml)	Unadjusted			Adjusted		
	*P-value*	OR	95% CI	*P-value*	OR	95% CI
ChAT	< 0.001	3.828	1.817 ~ 8.330	< 0.001	3.886	1.126 ~ 6.605
AChE	< 0.001	0.016	0.002 ~ 0.063	< 0.001	0.009	0.002 ~ 0.059
BuChE	< 0.001	0.001	0.000 ~ 0.009	< 0.001	0.003	0.000 ~ 0.008

### Receiver operating characteristic (ROC) curve results of AChE, BuChE and ChAT Concentrations and activities in CSF

**Fig. 5:**
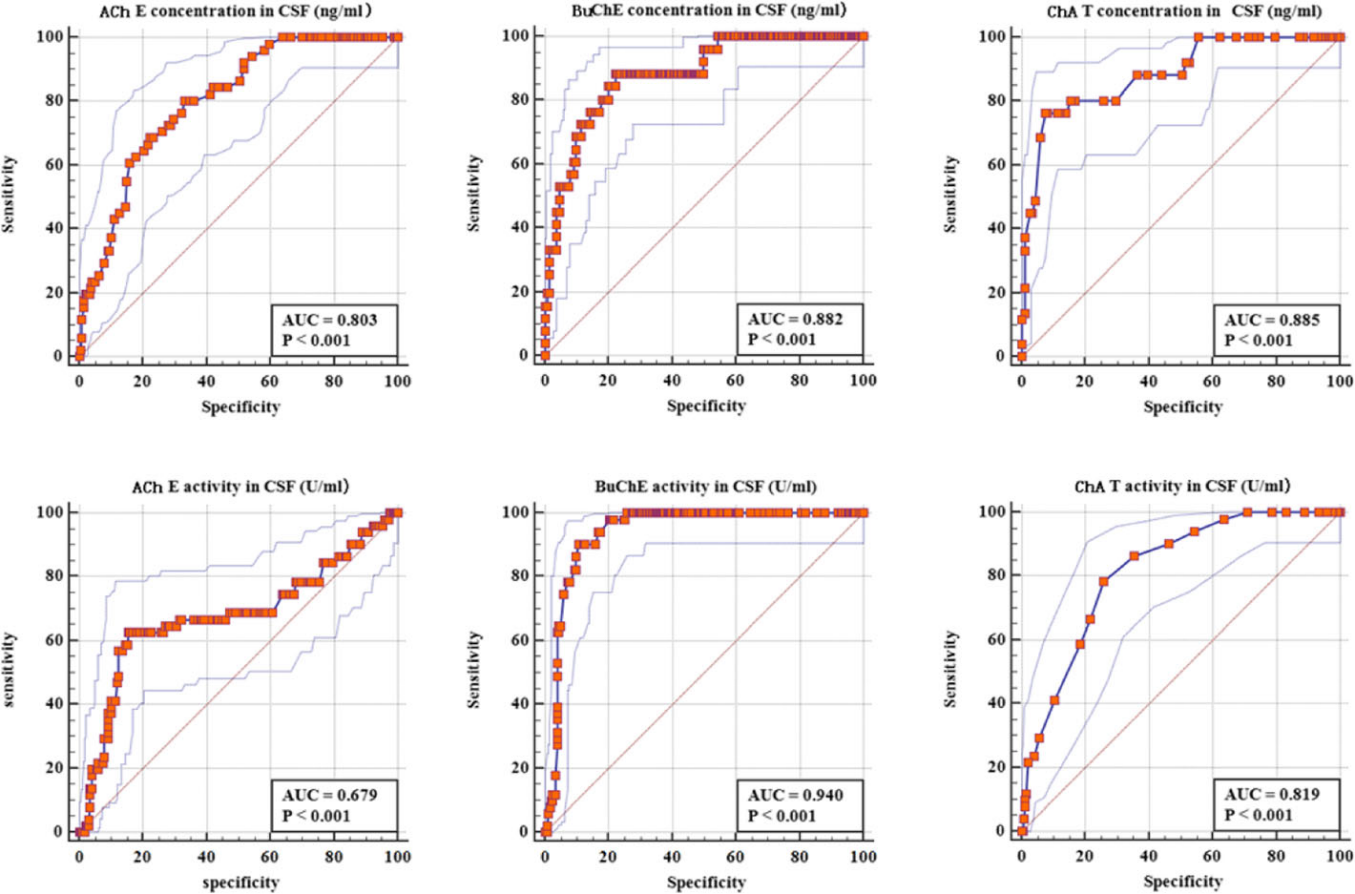
The ROC curve analysis of AChE, BuChE and ChAT showed that the activities and concentrations of AChE, BuChE and ChAT had high diagnostic value for POD, among which BuChE activity had the greatest diagnostic value, with all the AUCs greater than 0.5 and close to 1.0

The ROC curve analysis of AChE, BuChE and ChAT showed that the activities and concentrations of AChE, BuChE and ChAT had high diagnostic value for POD, among which BuChE activity had the most accurate diagnostic value, with all the AUCs greater than 0.5 and close to 1.0. (Table 3, Fig. 5).

**Table 3:** The ROC curve analysis of AChE, BuChE and ChAT showed that the activities and concentrations of AChE, BuChE and ChAT had high diagnostic value for POD, among which BuChE activity had the greatest diagnostic value, with all the AUCs greater than 0.5 and close to 1.0

CSF’s indexes	AUC	95% CI(L)	95% CI(U)	Youden’s index	Sensitivity	Specificity
AChE’s concentration (ng/ml)	0.803	0.763	0.839	0.4723	0.804	0.668
BuChE’s concentration (ng/ml)	0.882	0.848	0.910	0.6604	0.882	0.778
ChAT’s concentration (ng/ml)	0.885	0.852	0.913	0.6899	0.765	0.925
AChE’s activity (U/ml)	0.685	0.634	0.722	0.4728	0.628	0.845
BuChE’s activity(U/ml)	0.940	0.914	0.960	0.7947	0.902	0.893
ChAT’s activity (U/ml)	0.819	0.780	0.853	0.5250	0.784	0.741

## Discussion

In this study, we assessed the associations of POD with concentrations of AChE, BuChE, ChAT, TNF-α and IL-6 and activities of AChE, BuChE and ChAT in preoperative CSF and preoperative and postoperative plasma among 447 older adults who underwent total hip and knee replacement under spinal-epidural joint block. We found decreased concentration and activity of AChE and BuChE and increased concentration and activity of ChAT as well as increased concentrations of IL-6 and TNF-α in preoperative CSF and preoperative and postoperative plasma were observed in patients who developed POD, with BuChE in preoperative CSF having the most significant differences. The ROC curve analysis of AChE, BuChE and ChAT concentrations in CSF showed that BuChE activity had the most diagnostic value. Taken together, these findings suggested that activities of AChE, BuChE and ChAT in preoperative CSF might be associated with the neuropathology of POD, pending further investigation.

The incidence of POD in our study was 11.4%, which was consistent with the previous results of 3.6–41% [3, 20, 21]. For instance, previous studies have shown that the incidence of POD after total knee and hip replacement under spine anesthesia is 20% [21]. It is known that the incidence of POD is related to age, perioperative factors and postoperative pain [22] and sleep disorders [23]. Therefore, our research adopts CAM method [11, 12] to improve the accuracy of assessments of POD.

Nowadays, cholinergic biomarkers for POD are still largely unknown and notably, and the early stage of POD could not be effectively predicted and diagnosed [4]. The cholinergic theory greatly contributes to the pathogenesis of POD. CAIP is a neural circuit that affects and regulates inflammatory response. ACh is released by stimulating the efferent pathway of vagus nerve. ACh interacts with α7nAChR, which can inhibit the occurrence of Alzheimer’s disease (AD) [24]. ACh is an important neurotransmitter, is dynamically balanced by ChAT and counteractive cholinesterase (ChE) (including AChE and BuChE). Importantly, ChAT and ChE activities in CSF and nicotine binding sites could be used as important cognitive measurements, and the study found that the ratio of ChAT to ChE in AD patients increased [6]. Therefore, we speculate that AChE, BuChE and ChAT are involved in the changes of cognitive function.

In addition, the plasma ChE activity in POD patients decreased significantly before operation, indicating the changed ChE in resting state was a risk marker for POD [18]. Accumulating evidence shows that the degeneration of central cholinergic neurons is crucial in the onset of POD. The central cholinergic nervous system plays an important role in the formation and maintenance of learning and memory. Studies have found that after 40 years old, the central cholinergic neurons of human beings begin to degenerate. Different individuals have different degrees of degeneration [25]. At the same time, a study has shown that degeneration of the central cholinergic nervous system will lead to the occurrence of AD, and CSF analysis has confirmed that the elderly with AD will have increased risk of POD [5]. The changes of plasma cholinergic biomarkers in patients are related to the occurrence and development of POD [19]. The above studies suggest that degeneration of central cholinergic nervous system may be an important reason for POD.

So far, few of POD studies have focused on animal studies, and animal studies are difficult to fully simulate clinical phenotypes. Some studies have found that CSF biomarkers have more clinical significance and greater diagnostic value than those in plasma [7]. Based on the existing literature results, we assume that CSF cholinergic biomarkers have close associations with the occurrence and development of POD in elderly patients.

Studies have proved that CAIP is a neural circuit that affects and regulates inflammatory response. Stimulating the CAIP of the vagus nerve to release Ach can inhibit the release of inflammatory factors, such as IL-6 and TNF-α [26]. Moreover, α7nAChR mediates central anti-inflammatory response, by effectively activating the CAIP, and inhibiting the release of TNF-α and IL-6, leading to improvement cognition. The finding in preclinical models that AChE exerts neuroprotective effects mediated by α7nAChR and modulates innate immunity, possibly as a result of the increased availability of acetylcholine activating the CAIP, paves the way for new pharmacological intervention in AD and other neurological disorders characterized by neuroinflammation [5]. Activated inflammatory cells and inflammatory factors, especially IL-6 and TNF-α, can enter the central nervous system through damaged blood-brain barrier or periventricular organs to activate microglia. Changes in permeability may create conditions for the early occurrence of POD [27, 28]. Therefore, in this study, the detection of IL-6 and TNF-α before and after operation will further verify the occurrence of POD in patients.

In this study, the Spearman correlation analysis showed that concentrations and activities of AChE, BuChE, ChAT, TNF-α and IL-6 in plasma and CSF had good correlations. The approximate changes of CSF after operation can be estimated according to the changes of plasma before and after operation, because we know that it is impossible to extract the relevant indexes of CSF after operation following medical ethics.

Compared with patients without POD, the concentrations and activities of AChE and BuChE in CSF of patients with POD decreased significantly before operation, especially BuChE, and the concentration and activity of ChAT increased significantly, which indicated that degeneration of central cholinergic nervous system was involved in the development of POD. In addition, compared with patients without POD, the concentrations of IL-6 and TNF-α in CSF of patients with POD were significantly increased before operation, which indicated that the increased expression of inflammatory factors facilitated the occurrence and development of POD.

In our study, logistic regression was used to analyze the correlation of POD with the activities of ChAT, AChE and BuChE in CSF, and to adjust for the influencing factors such as sex, age, years of education, height, weight, body mass index (BMI), and ASA class. ChAT activity *P* value in CSF was less than 0.001, the adjusted OR value was 3.886, and the 95%CI confidence interval was 1.126 ~ 6.605. The OR value of ChAT activity is greater than 1 before and after adjustment, which indicates that adjusted ChAT activity is positively correlated with POD, that is to say, high ChAT activity in preoperative CSF after adjustment is still associated with POD. The *P* values of AChE and BuChE activity in CSF are less than 0.001, AChE adjusted OR value of AChE activity is 0.009, the 95%CI confidence interval is 0.002–0.059, the adjusted or value of BuChE activity is 0.003, and the 95%CI confidence interval is 0.000–0.008. The OR values of AChE and BuChE activities are less than 1 before and after adjustment, indicating that activities of AChE and BuChE after adjustment are negatively correlated with POD, in other words, low activities of AChE and BuChE in preoperative CSF after adjustment are still associated with POD. This result is basically consistent with the previous research results using plasma to detect cholinergic biomarkers [18].

The ROC curve analysis of AChE, BuChE and ChAT concentrations and activities in CSF showed that BuChE activity had the greatest diagnostic value. Therefore, high ChAT activity, low activities of AChE and BuChE can predict the occurrence and development of POD before operation, with BuChE as the focus. The decrease in plasma BuChE activity, and its influence have been investigated in diseases with a cholinergic deficit such as AD, and the reduction of total plasma BuChE activity is probably associated with a feedback mechanism providing a perspective for using BuChE as a possible plasmatic marker for AD [29]. At the same time, our study shows that AChE, BuChE and ChAT activities have good correlation between plasma and CSF. Therefore, our results show that low BuChE activity in preoperative CSF can predict the occurrence and development of POD. It is the future direction of our research to prove the findings in animal experiments and explore the relevant mechanisms.

There were limitations in our research. First, due to the small sample size, our study could not fully prove the role of CSF cholinergic biomarkers in predicting the occurrence and development of POD. In the future, the result will be replicated in our on-going large-scale study, which has been supported by the National Natural Science Foundation of China (No.91849126), and 1629 patients have been included.) Second, after the operation, we only evaluated POD once a day. Maybe twice a day will increase the accuracy of assessment of POD incidence. Third, it is well known that the CSF cholinergic biomarkers play an important role in neurodegenerative diseases. However, our research only focuses on AChE, BuChE and ChAT. There might be more cholinergic biomarkers, such as vesicular acetylcholine transporter (VAchT) and choline transporter (CHT), which could contribute to the neuropathology of POD [10]. We hope that more CSF cholinergic biomarkers can be detected for predicting the occurrence and development of POD in the future.

## Conclusion

CSF cholinergic biomarkers are associated with postoperative delirium in elderly patients, with BuChE activity having the closest association, which may be related to central cholinergic degeneration. To date, it remains unknown whether there is a causal relationship between activities of these cholinergic biomarkers and the development of POD. Hence, further studies are warranted.

## Supplementary information

**Additional file 1.**

## Data Availability

All data generated or analysed during this study are included in this published article and its supplementary information files.

## Abbreviations

POD: Postoperative Delirium
CSF: Cerebrospinal Fluid
MMSE: Mini-Mental State Examination
PACU: Post-anesthesia Care Unit
CAM: Confusion Assessment Method
ChAT: Choline Acetyltransferase
AChE: Acetylcholinesterase
BuChE: Butyrylcholinesterase
IL-6: Lnterleukin-6
TNF-α: Tumor Necrosis Tactor-α
ELISA: Enzyme-linked immunosorbent assay
BMI: Body Mass Index
ASA: American Society of Anesthesiologists
ROC: Receiver Operating Characteristic
CAIP: Cholinergic Anti-Inflammatory Pathway
Ach: Acetylcholine
α7nAChR: α7 nicotinic Acetylcholine Receptor
AD: Alzheimer’s Disease
VAS: Visual Analog Scale
EDTA: Ethylene Diamine Tetraacetic Acid.
ICC: Intraclass Correlation Coefficient
